# Comparison of total joint arthroplasty outcomes between renal transplant patients and dialysis patients—a meta-analysis and systematic review

**DOI:** 10.1186/s13018-020-02117-3

**Published:** 2020-12-09

**Authors:** Jiayi Li, Mingyang Li, Bo-qiang Peng, Rong Luo, Quan Chen, Xin Huang

**Affiliations:** 1 Department of Nephrology, the People’s Hospital of Dazu, Chongqing, 138#Longgang West Road, Longgang Street, Chongqing, 402360 China; 2 Department of Orthopaedic Surgery and National Clinical Research Center for Geriatrics, West China Hospital, Sichuan University, Chengdu, 610041 Sichuan Province China; 3 Department of Gastrointestinal Sursgery and Laboratory of Gastric Cancer, State Key Laboratory of Biotherapy, West China Hospital, Sichuan University and Collaborative Innovation Center for Biotherapy, Chengdu, 610041 China; 4 Department of Endocrinology, the People’s Hospital of Dazu, Chongqing, 138#Longgang West Road, Longgang Street, Chongqing, 402360 China

**Keywords:** Total joint arthroplasty, Renal transplant, Renal dialysis, Osteonecrosis of the femoral head

## Abstract

**Objectives:**

End-stage renal disease (ESRD) patients are at an increased risk of needing total joint arthroplasty (TJA); however, both dialysis and renal transplantation might be potential predictors of adverse TJA outcomes. For dialysis patients, the high risk of blood-borne infection and impaired muscular skeletal function are threats to implants’ survival, while for renal transplant patients, immunosuppression therapy is also a concern. There is still no high-level evidence in the published literature that has determined the best timing of TJA for ESRD patients.

**Methods:**

A literature search in MEDLINE, EMBASE, and the Cochrane Central Register of Controlled Trials (up to November 2019) was performed to collect studies comparing TJA outcomes between renal transplant and dialysis patients. Two reviewers independently conducted literature screening and quality assessments with the Newcastle-Ottawa Scale (NOS). After the data were extracted, statistical analyses were performed.

**Results:**

Compared with the dialysis group, a lower risk of mortality (RR = 0.56, Cl = [0.42, 0.73], *P* < 0.01, *I*^2^ = 49%) and revision (RR = 0.42, CI = [0.30, 0.59], *P* < 0.01, *I*^2^ = 43%) was detected in the renal transplant group. Different results of periprosthetic joint infection were shown in subgroups with different sample sizes. There was no significant difference in periprosthetic joint infection in the small-sample-size subgroup, while in the large-sample-size subgroup, renal transplant patients had significantly less risk (RR = 0.19, CI = [0.13, 0.23], *P* < 0.01, *I*^2^ = 0%). For dislocation, venous thromboembolic disease, and overall complications, there was no significant difference between the two groups.

**Conclusion:**

Total joint arthroplasty has better safety and outcomes in renal transplant patients than in dialysis patients. Therefore, delaying total joint arthroplasty in dialysis patients until renal transplantation has been performed would be a desirable option. The controversy among different studies might be partially accounted for that quite a few studies have a relatively small sample size to detect the difference between renal transplant patients and dialysis patients.

**Supplementary Information:**

**Supplementary information** accompanies this paper at 10.1186/s13018-020-02117-3.

## Introduction

Patients with end-stage renal disease (ESRD) are at an increased risk of osteonecrosis and osteoarthritis stemming from renal osteodystrophy, steroid use, amyloid deposition, and immunosuppressive therapy after renal transplant, which makes this population more likely to require total joint arthroplasty (TJA) [1–5]. However, the safety and post-operative outcomes of TJA are adversely affected by ESRD [6–8]. Dialysis and renal transplant are the most common therapeutic methods for ESRD patients; however, both methods might cause hazards for TJA. For dialysis patients, the high risk of blood-borne infection and impaired muscular skeletal function are threats to implants’ survival, while for renal transplant patients, immunosuppression therapy is also a concern [9].

Woods et al. [10] published the first report of a successful total hip arthroplasty (THA) in a renal transplant patient treated with cemented Charnley implants and without complications at 26 months of follow-up. Kenzora et al. [11] reported the first case series of THAs in renal transplant patients. The Harris hip scores improved from a mean of 45 to 100 postoperatively, without infection or aseptic loosening, up to 23 months after the operation. The first long-term follow-up study of THAs in renal transplant patients was reported by Cheng et al. [12]; with a minimum of 10 years of follow-up, they published that 78% of prostheses survived and good outcome scores were maintained with minimal complications. Naito et al. [13] first reported long-term results of THAs in dialysis patients, 35% (6/17) of the arthroplasties failed for loosening, and one patient died from an infection of the hip. Although many studies focused on TJA in ESRD patients, the case series results varied among the studies, regardless of whether dialysis patients or renal transplant patients were included. Even cohort studies that directly compared dialysis patients and renal transplant patients also presented conflicting results [14, 15]. In the International Consensus Meeting on orthopedic infection, patients with ESRD, who are also in need of TJA were discussed. The majority of experts supported that TJA should be performed after renal transplant, instead of replacing the joint while patients are on dialysis [16]. However, this recommendation was based on limited data.

The aim of this meta-analysis was to compare the rates of mortality, periprosthetic joint infection, revision, and postoperative complication between dialysis patients and renal transplant patients who underwent TJA. To our knowledge, no similar meta-analysis has been published to date.

## Methods

### Literature search

This meta-analysis followed the recommendations of PRISMA (the Preferred Reporting Items for Systematic Reviews and Meta-Analyses) [17] (Supplementary 1). A literature search in MEDLINE, EMBASE, and the Cochrane Central Register of Controlled Trials (all up to November 15, 2019) was systematically performed to obtain all original published articles focusing on comparing results of TJA in renal transplant or dialysis patients. We used “renal transplant”, “renal transplantation”, “kidney transplantation”, “kidney transplant”, “hemodialysis”, “haemodialysis”, “dialysis”, “HD”, “CAPD”, “arthroplasty”, “joint replacement”, “TKA”, “THA”, and “UKA” as the main retrieval terms. (The exact retrieval strategy is presented in Supplementary 2). After the screening, the reference lists of the included studies were manually examined.

### Selection criteria

The inclusion criteria were as follows: (1) focusing on the primary TJA, (2) selecting patients who are on dialysis and patients who underwent renal transplant, (3) with a cohort design, (4) providing available data for a meta-analysis of the outcomes we are interested in, and (5) including ≥ 10 patients. In contrast, studies that were incomplete or presented duplicate data were excluded.

Two reviewers screened all the records independently. Disagreements were resolved by discussion or the assistance of a third reviewer to reach a consensus. The reviewers examined the abstracts of the records from all sources and then filtered the studies on the basis of the selection criteria. Next, the full text of these studies was screened to confirm the eligibility of the studies.

### Quality assessment

Cochrane’s quality assessment tool was applied to evaluate the randomized controlled trials (RCTs) [18]. The cohort studies were assessed via the Newcastle-Ottawa Scale (NOS) [19]. Two reviewers independently assessed the quality of all included studies.

### Data extraction

Data was collected on the following two aspects: (1) Basic characteristics of the studies: author, country, year of publication, study design, database, site of surgery, sample size, dialysis type, follow-up period, etc. (2) The interested outcomes for meta-analysis: mortality, revision, peri-prosthetic joint infection, venous thromboembolic disease, dislocation, overall complications, and function score. Data extraction was performed by two reviewers independently, and disagreements were resolved by discussion or a third reviewer. We attempted to contact studies’ authors when missing or unclear data was encountered.

### Statistic analysis

Continuous variables were pooled by meta-analysis using the mean differences, which were considered significant when *P* values < 0.05 [20]. Heterogeneity between studies was assessed with the chi-squared test, where *P* < 0.1 and *I*^2^ > 50% indicated high heterogeneity. A fixed-effect model was applied when heterogeneity was not significant; otherwise, sensitivity analysis or subgroup analysis was conducted to investigate the potential source of heterogeneity. A funnel plot was used to evaluate the risk of publication bias in those studies. In studies where the exact number of integrated events was not presented, odds ratio and confidential interval in those studies were used to calculate an imputed value. Data analyses were performed using Review Manager version 5.3 software (Cochrane Foundation, McMaster University, Ontario, Canada).

## Results

**Fig. 1:**
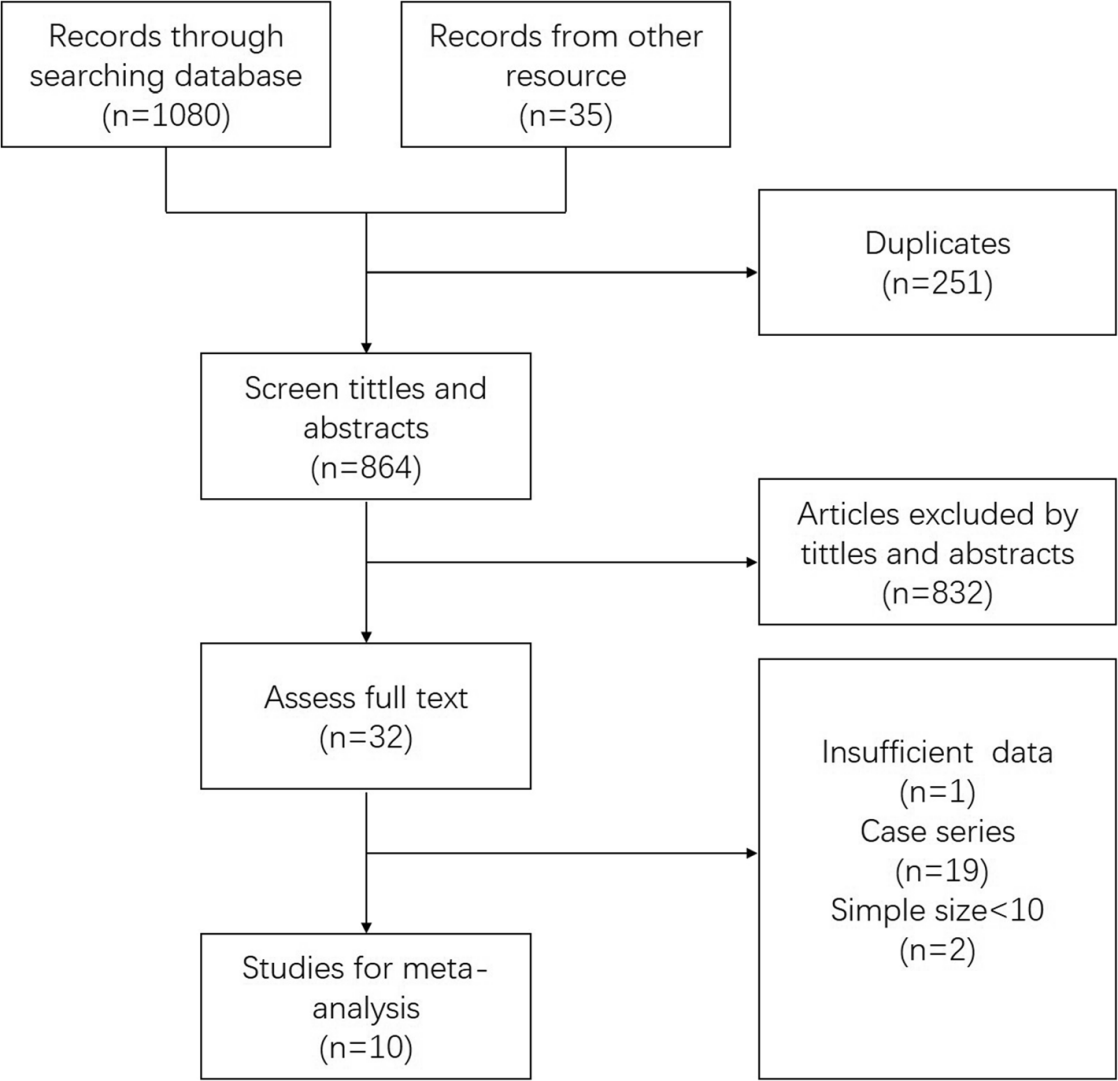
Flow chart of screening records

A total of 1080 records from the databases as mentioned earlier and 45 records obtained by manual retrieval were collected. Among them, 251 duplicates were deleted, and 832 records were removed according to the inclusion and exclusion criteria after examining their titles and abstracts. When the full text was examined, a total of 22 studies were filtered out because they were inappropriate for inclusion in the meta-analysis (one study did not report any of the outcomes that we were interested in, 19 studies were case series without a control group, and two studies enrolled fewer than ten patients). Ten studies [4, 9, 14, 15, 21–26] were ultimately included in this meta-analysis and systematic review (Fig. 1).

The included studies were assessed with the NOS scale, and the average score was 7.2 points, with no study scoring less than 6 points. Four studies [4, 9, 21, 25] did not clearly report the follow-up period. Six studies [14, 15, 21–23, 26] either did not report some important patient factors, such as age, or no solution was taken in their studies when there was a significant difference in demographics. Detailed scores for each study are presented in Supplementary 3.

Ten studies [4, 9, 14, 15, 21–26] with 6904 patients from four countries were included in this review, all of which were retrospective cohort studies published from 1995 to 2019. One study [23] included both total hip arthroplasties and hemiarthroplasties, and only the former were included in our meta-analysis. Total hip arthroplasties were studied in nine studies [4, 9, 14, 15, 21–24, 26], and total knee arthroplasties were studied in two studies [24, 25]. Four studies [4, 9, 21, 25] collected data from national databases, while the other six studies [14, 15, 22–24, 26] used data from the authors’ institutions with mid-term to long-term follow-up (from 44 to 132 months). Four studies [21, 23, 24, 26] clearly expressed that only hemodialysis patients were included in the dialysis group, but the other six studies [4, 9, 14, 15, 22, 25] did not provide information on whether peritoneal dialysis or hemodialysis was applied. In four studies [14, 15, 24, 26], patients in the renal transplant group had a younger average age than the dialysis group. Similarly, in another two studies [9, 21], there was a lower percentage of older patients in the renal transplant group (Table 1).

**Table 1:** Basic characteristics of included studies

Author, year of publication	Country	Joint	Data resource	Mode of dialysis	Fixation	Number of transplant patients (joints)	Number of dialysis patients (joints)	Follow-up (months)	Age of patients	Gender of patients (percentage of male, %)
Beau et al., 2017 [21]	USA	Hip	Medicare database	Hemodialysis	NA	902	2525	NA	NA	Transplant group: 60.4 Dialysis group: 48.7
Cavanaugh et al., 2016 [4]	USA	Hip/knee	Nationwide Inpatient Sample database	Dialysis	NA	1055	1747	NA	NA	NA
Debarge et al., 2007 [22]	France	Hip	Author’s institution	Dialysis	Both	14 (16)	14 (21)	72	56	57.1
Garcia et al., 2007 [23]	Spain	Hip	Author’s institution	Hemodialysis	13 cemented 6 cementless	8 (11)	7 (8)	44	56	38.9
Inoue et al., 2019 [24]	USA	Hip/knee	Author’s institution	Hemodialysis	Both	42 (57)	37 (50)	Transplant group: 62.9 Dialysis group: 72.5	Transplant group: 52.5 Dialysis group: 60.9	Transplant group: 50 Dialysis group: 59.5
Malkani et al., 2019 [9]	USA	Hip	Medicare database	Dialysis	NA	94	301	NA	NA	Transplant group: 44.7 Dialysis group: 49.8
Lieberman et al., 1995 [15]	USA	Hip	Three medical centers	Dialysis	Both	19 (30)	11 (16)	54	Transplant group: 35 Dialysis group: 51	Transplant group: 57.9 Dialysis group: 54.5
McCleery et al., 2010 [25]	UK	Knee	The Scottish Arthroplasty Project	Dialysis	NA	22	36	NA	NA	NA
Shrader et al., 2006 [14]	USA	Hip	Author’s institution	Dialysis	Cemented	28 (36)	9 (9)	Transplant group :132 Dialysis group: 72	Transplant group: 46 Dialysis group: 67	Transplant group: 53.6 Dialysis group: 77.8
Tornero et al., 2015 [26]	Spain	Hip	Author’s institution	Hemodialysis	Both	15 (18)	18 (20)	72	Transplant group: 53.8 Dialysis group: 75.0	Transplant group: 40.0 Dialysis group: 38.9

*NA* not available

### Primary outcomes

#### Mortality

**Fig. 2:**
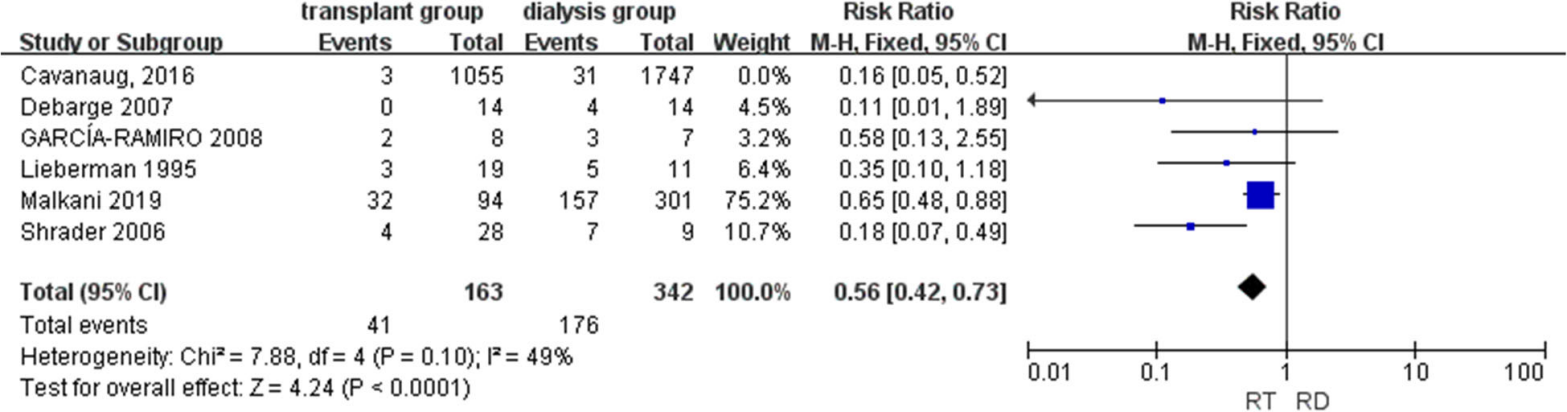
Forest plot of mortality (RT renal transplant, RD renal dialysis)

Mortality was reported in six studies, but the integrated result showed high heterogeneity (Supplementary 4). Therefore, a sensitivity analysis was performed, and it was found that Cavanaugh’s study [4] might be the potential source of the observed heterogeneity. The mortality reported in Cavanaugh’s study only included inpatient deaths, while other studies included all deaths. Therefore, Cavanaugh’s study was excluded from the meta-analysis of mortality. After excluding Cavanaugh’s study, the results of five studies [9, 14, 15, 22, 23] with a total of 505 patients demonstrated a lower risk of mortality in the renal transplant group than in the dialysis group (RR = 0.56, Cl = [0.42, 0.73], *P* < 0.01) with moderate heterogeneity (*I*^2^ = 49%) (Fig. 2).

#### Revision rate

**Fig. 3:**
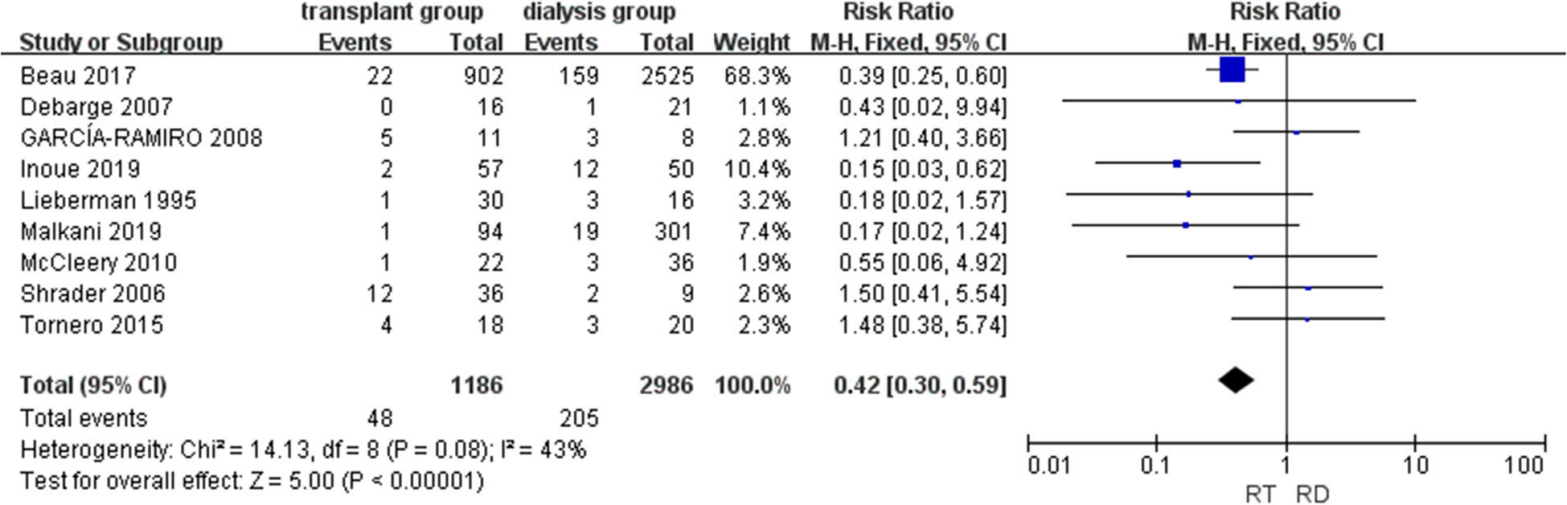
Forest plot of revision (RT renal transplant, RD renal dialysis)

Data on revision were presented in nine included studies [9, 14, 15, 21–26] involving 4172 joints. A lower risk of revision was shown in the renal transplant group in the meta-analysis (RR = 0.42, CI = [0.30, 0.59], *P* < 0.01), and the heterogeneity of the nine studies was acceptable (*I*^2^ = 43%) (Fig. 3).

#### Periprosthetic joint infection

**Fig. 4:**
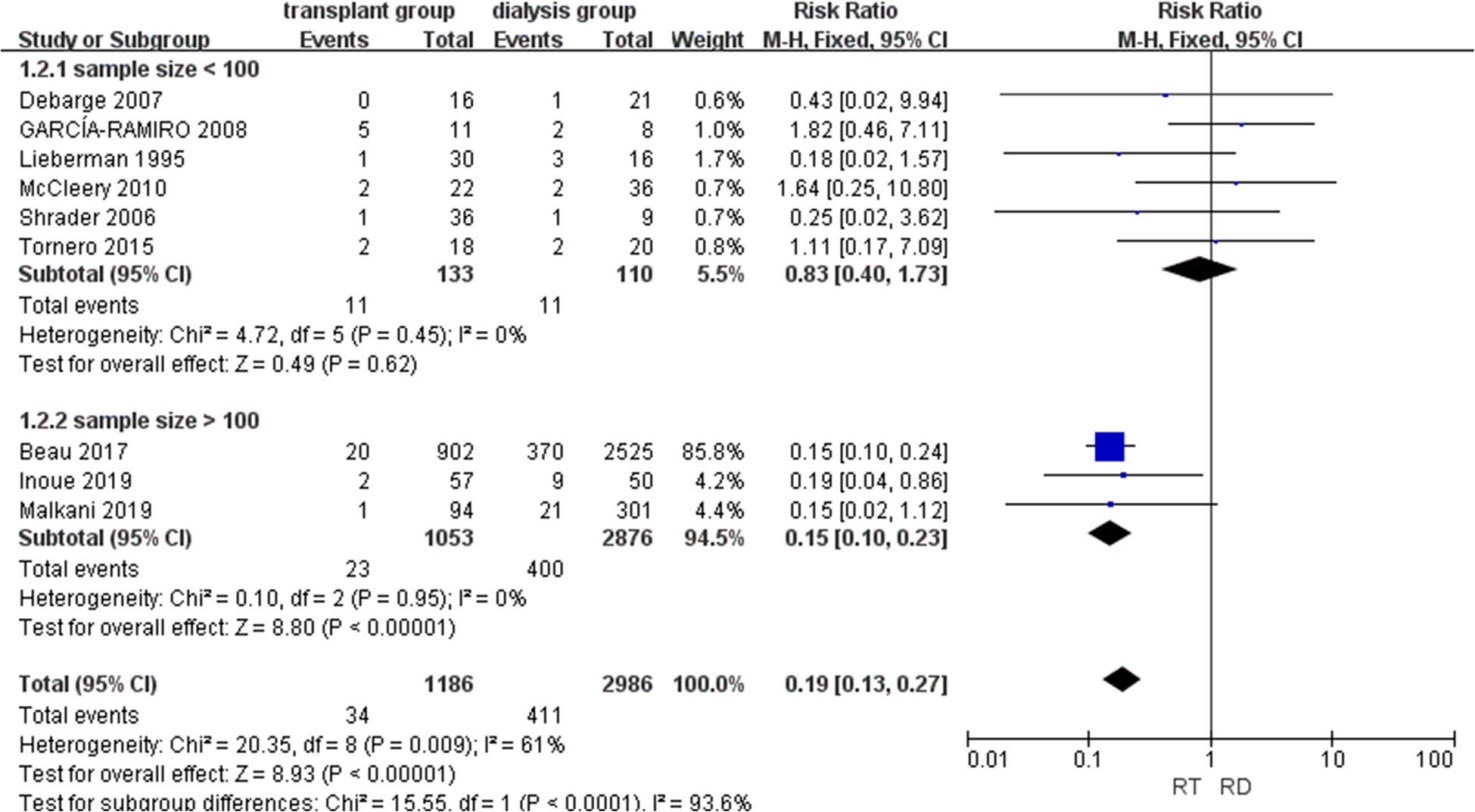
Forest plot of periprosthetic joint infection (RT renal transplant, RD renal dialysis)

In nine studies, 445 of the total 4172 joints were infected, and the overall heterogeneity was high (*I*^2^ = 61%). A subgroup analysis was conducted to reduce the heterogeneity and explore the potential source of heterogeneity. Studies with sample sizes larger than 100 were separated from those with sample sizes less than 100, and both subgroups had low heterogeneity. Six studies [14, 15, 22, 23, 25, 26] with 243 hips were included in the small-sample-size subgroup, and no significant difference in risk of infection was detected between the renal transplant group and dialysis group (RR = 0.83, CI = [0.40, 1.73], *P* = 0.62, *I*^2^ = 0%). In contrast, in the large-sample-size subgroup, which involved three studies [9, 21, 24] and 3929 joints, significantly lower risk of infection was shown in the renal transplant group (RR = 0.19, CI = [0.13, 0.23], *P* < 0.01, *I*^2^ = 0%) (Fig. 4).

### Secondary outcomes

**Fig. 5:**
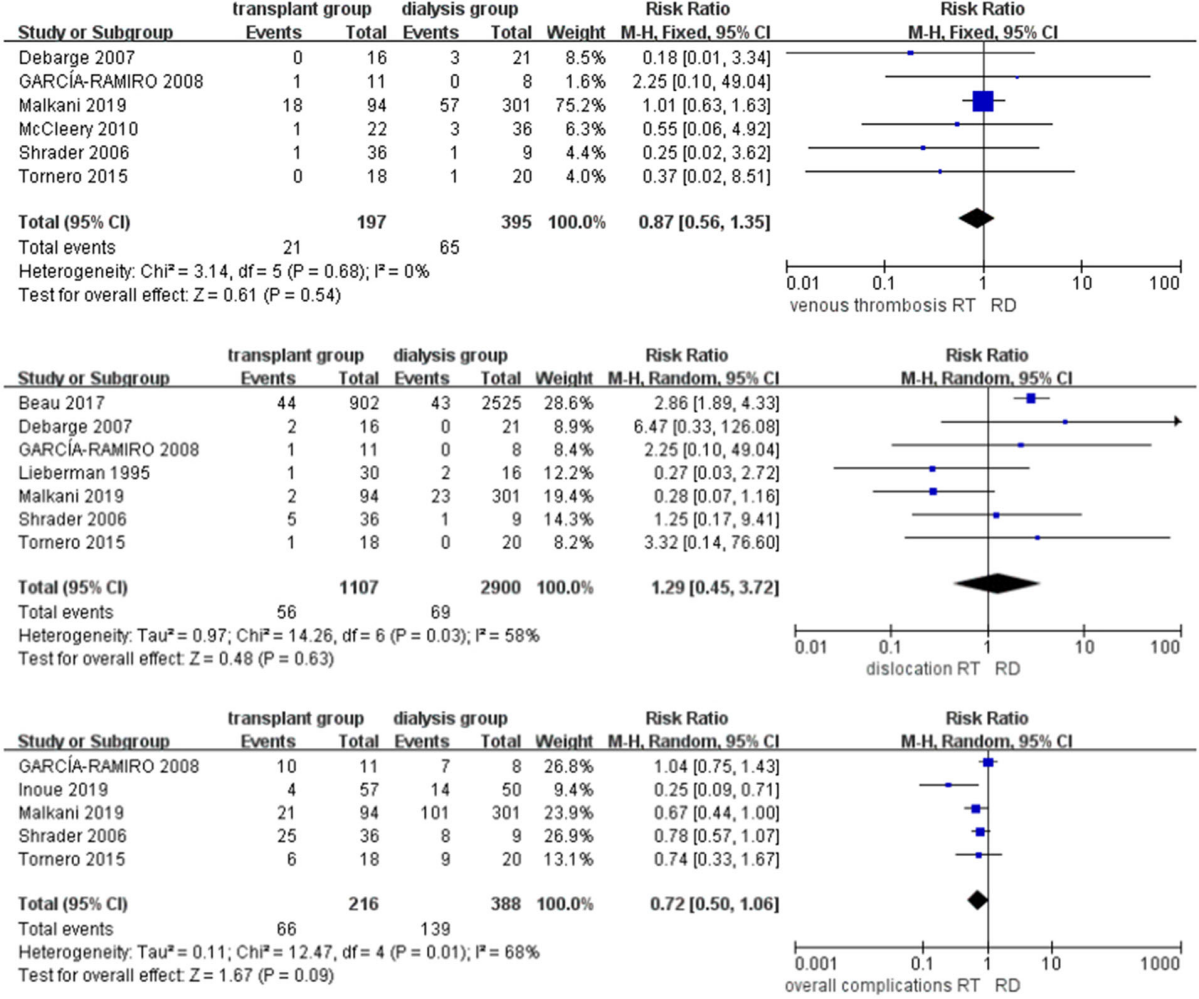
Forest plots of secondary outcomes (RT renal transplant, RD renal dialysis)

Overall complications were reported in five studies [9, 14, 23, 24, 26] with 604 joints. A random effect model was used to address the high heterogeneity, and the results revealed no significant difference in the risk of overall complications between the two groups (RR = 0.72, CI = [0.50, 1.06], *P* = 0.13). Similarly, there was no difference in the rate of dislocation and or venous thrombosis between the two groups. (RR = 1.29, CI = [0.45, 3.72], *P* = 0.63; RR = 0.87, CI = [0.56, 1.35], *P* = 0.54, respectively) (Fig. 5).

### Publication bias

**Fig. 6:**
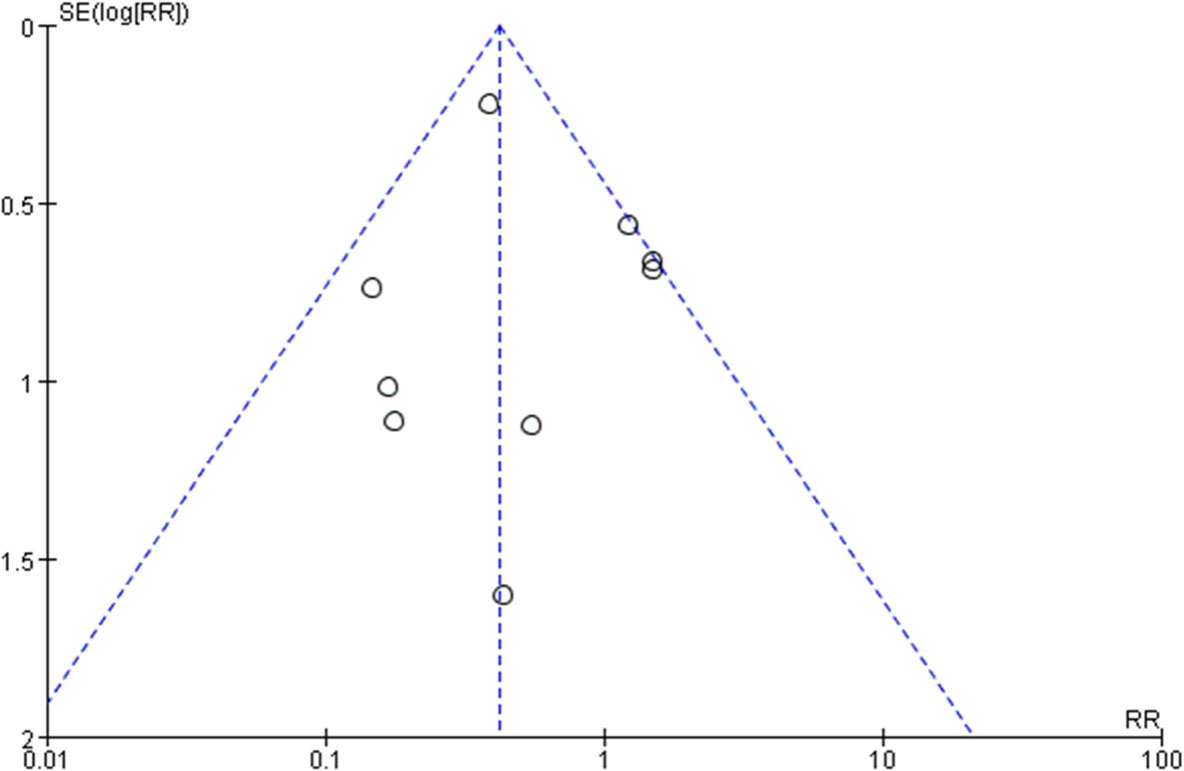
Funnel plot of revision

There was no apparent asymmetry in the funnel plot of revision rate, and it was inferred that a low risk of publication bias existed in those studies (Fig. 6).

## Discussion

This meta-analysis’s main finding is that arthroplasties performed in renal transplant patients have a lower risk of mortality, periprosthetic joint infection, and revision than those performed in dialysis patients. To our knowledge, this is the first meta-analysis on this topic, even though Lieu et al.’s [27] and Popat et al.’s [28] systematic reviews have been published. Unlike previous systematic reviews, this review provides a higher level of evidence due to the meta-analysis performed.

When Lieu and Popat conducted their systematic reviews, few cohort studies were published, and inadequate patients were included, making a meta-analysis challenging to achieve. Therefore, Lieu and Popat compared renal transplant and dialysis patients by directly adding data from different studies. Since case series without a control group is of inferior comparability, integrating results from different case series probably introduces high heterogeneity. Another limitation preventing the detection of the differences between dialysis patients and renal transplant patients in previous systematic reviews is the small sample size. A total of 755 joints and 797 joints were involved in the researches conducted by Lieu and Popat, respectively. As shown in our periprosthetic joint infection results, there was no significant difference between the two groups when only studies with small sample sizes (less than 100 patients) were evaluated; however, the difference was significant when extensive studies were evaluated. It seems that the sample size played an essential role in the conclusions from previous studies. Recently, arthroplasties in renal failure patients have recaptured surgeons’ attention, and several new and high-quality cohort studies [9, 24] focusing on this topic have been published; hence, we designed a meta-analysis only including cohort studies. Even though case series were excluded from our study, the overall number of included patients was much larger than in previous systematic reviews.

It is difficult to match the demographic characteristics of dialysis patients and renal transplant patients. Dialysis patients are relatively older and have more comorbidities. Some studies applied multivariate regression analysis to reduce the effect of confounding factors. Malkani et al. [9] conducted multivariate Cox regression analyses including patient factors (age, sex, comorbidity, Charlson index, diabetes, obesity, heart disease, census region, race, and socioeconomic status) and hospital factors (teaching status, ownership, year of surgery, location, and bed size). The results revealed that, compared with patients without renal disease, dialysis patients had a significantly higher risk of infection at 3 months, 6 months, 1 year, 2 years, and 5 years after surgery. Meanwhile, the data between renal transplant patients and patients without renal disease were not significant. A direct comparison was presented in the paper by Inoue et al. [24]. In brief, using logistic regression analysis, the authors concluded that, compared with patients on dialysis, renal transplant patients were less likely to have revision surgery. The results of the multivariate analysis supported that arthroplasties performed in renal transplant patients were more likely to achieve better clinical results than those performed in dialysis patients.

Due to long-term immunosuppression, periprosthetic joint infection is a significant concern for transplant patients. Several studies have presented an increased risk of infection in transplant patients. García et al. [23] reported that 5 hips of 11 THAs in renal transplant patients were infected in a follow-up of 3 years. Karas et al. [29] reported a 6% late infection rate in renal transplant patients. Alpert et al. [30] age-matched 24 transplant patients with 235 nontransplant patients and demonstrated a higher infection rate in the transplant group (3.7% vs 1.3%). Tannenbaum et al. [31] completed 35 joint replacements after renal or liver transplantation with an average follow-up of 8.8 years and reported that the infection risk was as high as 19%. Klatt et al. [32] also detected an infection rate of 17.3% in a similar patient population. However, more studies supported that the risk of infection in renal transplant patients was satisfactory. Some studies even claimed no significant difference between renal transplant patients and patients without renal disease regarding the rate of infection following TJA [9, 12, 21, 33–38]. Radford et al. [37] reported 31 THAs in 21 renal transplant recipients with an average follow-up of 6 years, and no infection was found. Lim et al. [36] compared 45 consecutive THAs in renal transplant patients with those in 96 sex-matched and age-matched patients without renal disease. No significant intergroup differences in infection were observed. This was also supported by the research of Malkani et al. [9], who performed multivariate Cox regressions, including patient factors and hospital factors. Although a consensus on the infection rate has not yet been reached, most authors have supported that renal transplant patients’ infection rate was acceptable and that TJA was a reasonable therapeutic option in those patients [12, 28, 30, 36].

It is difficult to demonstrate the underlying cause of the variation in outcomes from different studies. The type of surgery, method of fixation, and dialysis mode are all potential sources of heterogeneity. Palmisano et al. [39] reported an infection rate of 3.7% following total knee arthroplasties in transplant patients, which is higher than that in their THA group. The multivariate analysis from Inoue et al. [24] also revealed that, compared with THA, total knee arthroplasty was an independent risk factor for post-operative clinical complications (odds ratio, 3.964; *P* = 0.03). Due to inadequate bone stock, most studies have adopted cemented implants, and the results have been satisfactory. Some evidence suggests that cementless implants are also reliable in renal transplant patients [30, 40], and Popat et al. [28] concluded that cementless implants appeared to be associated with lower failure rates in both hemodialysis patients and renal transplant patients. However, long-term validation for cementless implants is still lacking and heterogeneity in Popat’s research is a concern. A recent study [41] demonstrated that the mode of dialysis is also essential; the hemodialysis patients have a significantly higher risk of infection, whereas patients on peritoneal dialysis do not appear to have a higher risk when compared with dialysis-independent patients.

There are several limitations of our meta-analysis. First, our meta-analysis was not able to provide advice about the method of fixation or the mode of dialysis, because only a few included studies reported data for those items. In addition, the timing of outcome measurements in the different included studies was inconsistent and not presented in some database studies. Additionally, no RCTs directly comparing arthroplasties in renal transplant patients and dialysis patients were found, and the number of cohort studies was not large. More high-quality studies on this subject need to be carried out in the future.

## Conclusion

The total joint arthroplasty has better safety and outcomes in renal transplant patients than in dialysis patients. Therefore, delaying total joint arthroplasty in dialysis patients until renal transplantation has been performed would be a desirable option. The controversy among different studies might be partially accounted for that quite a few studies have a relatively small sample size to detect the difference between renal transplant patients and dialysis patients.

## Supplementary Information

**Additional file 1.** Prisma 2009 Checklist.

**Additional file 2.** Retrieval strategy.

**Additional file 3.** Result of NOS.

**Additional file 4.** Plot of mortality before sensitive analysis.

## Data Availability

All data generated or analyzed during this study are included in this published article and its supplementary information files.

## Abbreviations

ESRD: End-stage renal disease
TJA: Total joint arthroplasty
THA: Total hip arthroplasty
TKA: Total knee arthroplasty
PRISMA: The Preferred Reporting Items for Systematic Reviews and Meta-Analyses
NOS: Newcastle-Ottawa Scale
RCTs: Randomized controlled trials
