# Supplementary material for: Comparison of total joint arthroplasty outcomes between renal transplant patients and dialysis patients—a meta-analysis and systematic review
Source: J Orthop Surg Res. 2020 Dec 9;15:590. doi: 10.1186/s13018-020-02117-3 (PMC7724818; doi:10.1186/s13018-020-02117-3)
Supplement: Supplementary file 2 — Additional file 2. Retrieval strategy. [file 13018_2020_2117_MOESM2_ESM.docx]

#1. (renal transplant or renal transplantation or kidney transplantation or renal transplantation).af.

#2. (hemodialysis or haemodialysis or dialysis or HD or CAPD).af.

#3. #1 OR #2

#4. (arthroplasty or joint replacement or TKA or THA or UKA).af.

#5. #3 AND #4
