# Supplementary material for: Comparison of total joint arthroplasty outcomes between renal transplant patients and dialysis patients—a meta-analysis and systematic review
Source: J Orthop Surg Res. 2020 Dec 9;15:590. doi: 10.1186/s13018-020-02117-3 (PMC7724818; doi:10.1186/s13018-020-02117-3)
Supplement: Supplementary file 3 — Additional file 3. Result of NOS. [file 13018_2020_2117_MOESM3_ESM.docx]

| Study | Selection | | | | Comparability control for important factor | Outcomes | | | Scores |
| --- | --- | --- | --- | --- | --- | --- | --- | --- | --- |
|  | Representativeness of the exposed cohort | Selection of the non exposed cohort | Ascertainment of exposure | Demonstration that outcome of interest was not present at start of study |  | Assessment of outcome | Follow-up long enough for outcomes to occur | Adequacy of follow up of cohorts |  |
| Postoperative Impact of Diabetes, Chronic Kidney Disease, Hemodialysis, and Renal Transplant After Total Hip Arthroplasty | ★ | ★ | ★ | ★ |  | ★ | ★ |  | 6 |
| Total hip arthroplasty in patients with renal failure | ★ | ★ | ★ | ★ |  | ★ | ★ | ★ | 7 |
| Total hip arthroplasty in hemodialysis and renal transplant patients | ★ | ★ | ★ | ★ |  | ★ | ★ | ★ | 7 |
| Comparison of Postoperative Complications and Survivorship of Total Hip and Knee Arthroplasty in Dialysis and Renal Transplantation Patients | ★ | ★ | ★ | ★ | ★★ | ★ | ★ | ★ | 9 |
| Complications and Readmission Incidence Following Total Hip Arthroplasty in Patients Who Have End-Stage Renal Failure | ★ | ★ | ★ | ★ | ★★ | ★ | ★ |  | 8 |
| Hip Arthroplasty in Patients with Chronic Renal Failure | ★ | ★ | ★ | ★ |  | ★ | ★ | ★ | 7 |
| Rates of infection and revision in patients with renal disease undergoing total knee replacement in Scotland | **★** | ★ | ★ | ★ | ★★ | ★ | ★ |  | 8 |
| Total Hip Arthroplasty in Patients With Renal Failure A Comparison Between Transplant and Dialysis Patients | ★ | ★ | ★ | ★ |  | ★ | ★ | ★ | 7 |
| Outcomes of hip arthroplasty in patients with end-stage renal disease: a retrospective, controlled study. | ★ | ★ | ★ | ★ |  | ★ | ★ | ★ | 7 |
| Complications and Mortality in Chronic Renal Failure Patients Undergoing Total Joint Arthroplasty: A Comparison Between Dialysis and Renal Transplant Patients | ★ | ★ | ★ | ★ |  | ★ | ★ |  | 6 |

A maximum of 2 stars can be allotted in this category, one for line of force, the other for other important factors
