# Supplementary figures and images for: Comparison of total joint arthroplasty outcomes between renal transplant patients and dialysis patients—a meta-analysis and systematic review
Source: J Orthop Surg Res. 2020 Dec 9;15:590. doi: 10.1186/s13018-020-02117-3 (PMC7724818; doi:10.1186/s13018-020-02117-3)

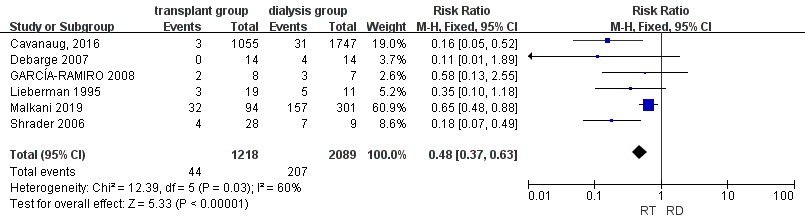

Supplement: Supplementary file 4 — Additional file 4. Plot of mortality before sensitive analysis. [file 13018_2020_2117_MOESM4_ESM.png]
